# 

**Published:** 1989-08

**Authors:** 


W1 BR323

.104 NO.3 1989

.D1--------SEQ: B33740000

I: BRISTOL MEDICO-CHIRURGICAL

JOURNAL

### BRISTOL MEDICO-CHIRURGICAL JOURNAL

VOLUME 104(iii) AUGUST 1989

### RICHARD BRIGHT COMMEMORATIVE NUMBER

**Richard Bright—a man of many parts**
**Alternative approach to sex offenders**
**Hyperparathyroidism**
**GPs and Consultants**
**Tracheo-bronchial foreign bodies**
**Rural Practice**
**Shakespeare—a new discovery**
**Psychopathology in Art**
**With Burton in India—final episode**
**Reports from Societies, etc.**

NATIONAL LIBRARY
FEB 02 1990
OF MEDICINE

THE MEDICAL JOURNAL OF THE SOUTH WEST

